# Cardiac Autonomic Neuropathy Measured by Heart Rate Variability and Markers of Subclinical Atherosclerosis in Early Type 2 Diabetes

**DOI:** 10.5402/2012/168264

**Published:** 2012-12-04

**Authors:** Hossein Fakhrzadeh, Ahmad Yamini-Sharif, Farshad Sharifi, Yaser Tajalizadekhoob, Mojde Mirarefin, Maryam Mohammadzadeh, Saeed Sadeghian, Zohre Badamchizadeh, Bagher Larijani

**Affiliations:** ^1^Elderly Health Research Center, Endocrinology & Metabolism Research Center, Tehran University of Medical Sciences, Dr Shariati University Hospital, North Kargar Avenue, Tehran, Iran; ^2^Tehran Heart Center, Tehran University of Medical Sciences, North Kargar Avenue, Tehran, Iran; ^3^Endocrinology & Metabolism Research Center, Tehran University of Medical Sciences, Tehran, Iran

## Abstract

Cardiac autonomic neuropathy (CAN) is a critical complication of type 2 diabetes mellitus (T2DM). Heart rate variability (HRV) is a noninvasive tool to assess cardiac autonomic function. We aimed to evaluate whether CAN is associated with increased risk of atherosclerosis in T2DM. A total of 57 diabetic and 54 nondiabetic subjects, free of coronary heart disease, were recruited. Carotid intima media thickness (CIMT), coronary calcium score (CAC), and brachial Flow Mediated Dilation (FMD) were measured. Heart rate variability and vagal components of autonomic function were determined. Significant reduction of normalized HF power (*P* < 0.05) and total power (*P* < 0.01) was observed in T2DM. CIMT and CAC scores were significantly higher while FMD was significantly lower in diabetics (*P* < 0.01 for all). Median HbA_1c_ levels were significantly higher in diabetics. CIMT was inversely and independently associated with total power both in diabetics and controls (*P* < 0.01 for both groups). There was also an inverse association between total power and median HbA_1c_. Autonomic dysfunction, especially parasympathetic neuropathy, was present since early-stage T2DM. This was related to subclinical atherosclerosis. Early detection of cardiac autonomic neuropathy can help us detect the development of atherosclerosis earlier in T2DM to prevent unfavorable outcomes.

## 1. Introduction

Cardiac autonomic neuropathy (CAN) is a serious complication of type 2 diabetes mellitus (T2DM) which carries an approximately 5-fold increased risk of mortality in these patients [1, 2]. Damage to the autonomic innervations of the heart is a harbinger of dire outcomes such as lethal arrhythmias and sudden cardiac death [3].

Heart rate variability (HRV) analysis is a noninvasive tool to assess cardiac autonomic function. The value of HRV is that it can detect CAN before conventional tests of cardiovascular autonomic function like the Ewing battery [4–6].

In the Framingham Heart Study, both frequency and time domain measures of HRV were found to be inversely associated with the risk of mortality [7]. In the Atherosclerosis Risk In Communities (ARIC) Study, decreased HRV was independently associated with the risk of developing coronary heart disease [8, 9]. 

On the other hand, T2DM is associated with accelerated atherosclerosis, with markedly increased incidence of cardiovascular morbidity and mortality [10].

Thus, early detection of atherosclerosis is of utmost importance in T2DM to prevent negative outcomes. 

Framingham Risk Score has been proved useful for detection of coronary heart disease risk before its serious negative end points become apparent [11, 12]. Notwithstanding, due to limitations of this scoring system surrogate markers of atherosclerosis have been devised to enhance prevention and earlier diagnosis of coronary heart disease in T2DM [13, 14].

We performed this study to evaluate whether CAN as measured by decreased HRV is associated with increased risk of subclinical atherosclerosis in T2DM.

## 2. Materials and Methods

### 2.1. Subjects

This case-control study was performed in Dr. Shariati hospital, Tehran University of Medical Sciences. A total of 57 diabetic and 54 nondiabetic control subjects were recruited in this study. Participants were drawn from the list of diabetics who attended diabetes outpatient clinics in Dr. Shariati Hospital, Tehran, Iran, for regular followup between September 2010 and January 2011. The control group was healthy relatives in law of diabetic participants. The study protocol was approved by the ethics committee of Tehran University of Medical Sciences, and the participants signed their informed consent at the time of recruitment.

The inclusion criteria were defined as both sexes, aged between 30 and 65 years. Current or previous smokers, subjects with diabetic foot, renal failure (GFR < 90), history of malignancy, and cirrhosis were excluded from the study. In addition, none of the participants had clinical coronary heart disease as evidenced by Rose questionnaire and electrocardiographic (ECG) criteria (Minnesota codes 1.1–1.3, 4.1–4.4, 5.1–5.3, and 7.1) at the time of the study.

Patients with proliferative retinopathy were excluded from the study according to their results of retinoscopy which was performed by two ophthalmologists. Peripheral neuropathy was diagnosed using the10-gram Semmes-Weinstein monofilament test on the plantar foot surface in diabetic patients. Failure to sense the filament was considered as diabetic neuropathy and resulted in exclusion from the study.

Height was measured with a Stadiometer, and weight was assessed by a calibrated beam balance. Body mass index (BMI) was calculated as weight (Kg) divided by height (M) squared. Blood pressure was measured twice (5 minutes apart) using a standard calibrated mercury sphygmomanometer on both right and left arms after the participants had been sitting calm for at least 10 minutes. The highest blood pressure of two sides was considered as participant's blood pressure. The two groups were matched based on age. 

#### 2.1.1. Definitions

Diabetes mellitus was defined as patients with FBS ≥ 126 mg/dL, or 2-h postload glucose ≥200 mg/dL or else were using oral hypoglycemic agents [14]. Hypertensive patients were those with systolic blood pressure of ≥ than 140 or diastolic blood pressure of ≥90 mmHg or were using antihypertensive drugs [15].

### 2.2. Laboratory Data

Venous blood samples were collected in the morning after 12 h fasting. The blood samples were centrifuged, and then serum was collected for measuring the biomedical parameters. 

In all the participants, fasting plasma glucose (FBS) and 2-h postload glucose levels were measured. 

Plasma levels of glucose, triglyceride (TG), total cholesterol, HDL cholesterol, LDL cholesterol, creatinine, blood urea nitrogen (BUN), and highly sensitive C-reactive Protein (hs-CRP) were measured by a colorimetric method using Pars Azmoon kit with an autoanalyzer (Hitachi 902, Boehringer Mannheim Germany). Serum Insulin concentration was assessed by immunoassay (ELISA) using a Bioscience kit (Monobind kit, USA). HbA_1c_ was detected by High-performance liquid chromatography (HPLC) (Knauer, Germany), coupled with fluorescence detector. The method was validated over a linearity range of 1–100 *μ*mol/L of the plasma. The intra- and interassay coefficients of variation (CVs) for all these measurements were <4%, which was less than allowed CVs. A morning clean catch midstream urine sample was collected for measuring creatinine and evaluation of microalbuminuria. 

Homeostasis model assessment (HOMA) index was calculated as the product of the fasting plasma insulin level (*μ*IU/mL) and the fasting plasma glucose level (mmol/L), divided by 22.5.

#### 2.2.1. Surrogate Atherosclerosis Markers

We measured the following markers of subclinical atherosclerosis (SCA) in our participants.

**Carotid Intima Media Thickness (CIMT)**

Ultrasonographic analysis of the carotid artery was performed with a high-resolution ultrasound scanner, equipped with a linear array 13 MHz transducer (MyLab 70 XVision, Biosound Esaote, USA). One physician who was blind to the nature of the group performed CIMT measurements. A rapid cross sectional scanning was made in the first step to pinpoint the possible plaques. The scan was started from the proximal part of the common carotid artery (CCA) toward the bifurcation, followed by scanning the internal and then the external carotid arteries. This process was followed by a longitudinal scanning of the CCA. In this step, the dynamic sequence images were stored for the following measurements of CIMT. A segment of the artery (usually where the vessel walls were most clearly seen throughout the recording) was magnified to identify a distinct lumen-intima and media-adventitia interface. CIMT was defined as the distance between the leading edge of the lumen-intima interface and the leading edge of the media-adventitia interface. For detection of CIMT, a special software (Vascular tools 5 (Medical Imaging Applications LLC, USA)) was employed. The regions of interest were defined as 1.0 cm distal to the bifurcation, the bifurcation and 1.0 cm proximal to the internal carotid artery in both near and far walls. Then for each subject CIMT was reported as the average of 12 measurements (6 measurements from the right and 6 from the left carotid artery).

**Coronary Artery Calcium Score (CAC)**

Anterior-posterior and lateral chest scout views were first obtained for planning. Calcium score images were obtained with a Phillips 64 MDCT scanner using 64 × 2.5 mm × 400 ms with 120 KVP and 50–75 mAs to cover the entire heart and proximal ascending aorta. A positive calcium score was defined by 130 HU with an area of 1 mm^2^ or greater. The amount of calcium was quantified using the Agatston scoring method [16].

**Flow-Mediated Dilation (FMD)**

FMD of the brachial artery was measured according to the American College of Cardiology guidelines [17]. The diameter of the right brachial artery was measured 3–5 cm above the antecubital fossa. Then a blood pressure cuff was inflated around the right forearm to at least 50 mmHg above the systemic blood pressure for 4-5 minutes. 60 seconds after cuff release, the diameter of the brachial artery was measured again. The brachial FMD was calculated as the percentage of change in the maximum postocclusion diameter of the brachial artery relative to the mean baseline diameter. All the measurements were performed in the end-diastolic phase coinciding with the R-wave on an electrocardiograph monitor. Every measurement was taken as the average of 3 consecutive cardiac cycles. FMD values greater than 5–10% were considered normal [17].

**Heart Rate Variability**

The evaluation of HRV was performed in a quiet and temperature-controlled room according to the guidelines of the Task Force for Pacing and Electrophysiology [18]. Participants were advised to abstain from caffeinated food and beverages on the day of their assessments. Repeat assessments were performed at precisely the same time of day after 48 hours. After 15 minutes of supine rest with a regular and calm breathing pattern, a continuous 10-minute ECG recording was collected using an applanation tonometer interface with HRV software (SphygmoCor, AtCor Medical Pty, Sydney, Australia). The high-frequency (HF band: 0.15–0.45 Hz), low-frequency (LF band: 0.04–0.15 Hz), and very-low-frequency (VLF band: 0.01–0.04 Hz) components of HRV (measured in absolute units; i.e., ms^2^) were obtained. Total power (TP) of HRV was also calculated to be used in regression analysis as a global marker of cardiac autonomic function. Normalized HF and LF powers were determined by dividing their absolute powers by the total power minus the VLF component and multiplied by 100 [18–20]. From the electrocardiographic recording, the following statistical and geometric time domain indices were calculated from RR intervals: standard deviation of the NN intervals (SDNN), the square root of the mean squared difference of successive NNs (RMSSD), and the triangular index (TI). Frequency domain variables including total, HF, and LF powers and LF : HF ratio were derived from spectral analysis of successive R-R intervals [18].

**Assessment of Cardiac Autonomic Function**

HRV measurement was performed after Valsalva and standing maneuvers in addition to supine state, using SphygmoCor software (SphygmoCor, AtCor Medical Pty, Sydney, Australia).

For valsalva maneuver, the participant was requested to blow into the mouthpiece of the device manometer to a pressure of 40 mmHg for 15 seconds. Then the valsalva ratio was calculated as the relationship between the longest and shortest R-R intervals after strain. For standing maneuver, the participant was requested to breathe at a normal pace for 5 minutes in the supine state. Then he/she was asked to go from supine to a full upright position and remain standing until the end of measurement. The standing ratio was calculated as longest R-R interval around the 30th beat after standing up to the shortest R-R interval around the 15th beat during standing.

We also performed deep breathing test by calculating the ratio of maximum and minimum heart rates during six cycles of paced deep breathing (E/I index) [21].

### 2.3. Data Analysis

For data analysis, the SPSS (version 18.0) was used. Normality of data distribution was evaluated by the Kolmogorov-Smirnov test. For data not normally distributed, the Mann-Whitney *U* test was applied. TP, LFnorm, HFnorm, LF : HF, RMSSD, and valsalva ratio were log-transformed before analysis due to their nonnormal distribution. For comparing data with normal distribution, unpaired *t*-test was used. Adjustment for confounding factors was performed using univariate analysis of variance (ANOVA). Correlation of variables was demonstrated using Pearson's and Spearman's correlation coefficients in normal distributed parametric and nonparametric variables, respectively. For assessment of association of variables, linear regression and logistic regression were used for parametric and binary variables, respectively. 

## 3. Results

Table 1 summarizes the general characteristics and subclinical atherosclerosis markers of the two groups. Mean duration of diabetes was 8.6 years in T2DM participants. Systolic blood pressure was significantly higher and HDL-C levels were significantly lower in diabetics. Total- and LDL-cholesterols were significantly higher in diabetics. Fasting insulin and). HbA_1c_ levels were significantly higher in diabetics than in controls. CIMT and CAC scores were significantly higher, while brachial FMD was significantly lower in diabetics.

Table 2 compares the autonomic and HRV indices between T2DM and control groups. SDNN and RMSSD were significantly lower in diabetics than in controls. Significant reduction of spectral power in HF band (expressed as normalized units) and in total power was also observed in our T2DM participants relative to controls.

Valsalva ratio, E/I index and heart rate response to standing were significantly lower in diabetics relative to controls.

HF power was highly correlated to total power. In addition, the observed associations for total power were similar to those of HF power. So we performed our analyses with total power for convenience.

Unadjusted correlations between total power and conventional CHD risk factors revealed that diastolic BP in all participants and BMI in diabetics were negatively associated with total power (Table 3). 

Multiple logistic regression of the association between surrogate atherosclerosis markers and total power spectra revealed that CIMT was inversely and independently associated with total power both in diabetics and controls (Table 4). This relationship between total power and CIMT showed a dose-response pattern throughout the distribution of HRV. On the other hand, although CAC score also was inversely associated with HRV in a stepwise manner, this relation lost its significance after adjustment for diabetes (Table 2). The relationship between FMD% and total power was lost after multiple regression (Table 4).

**Figure 1:**
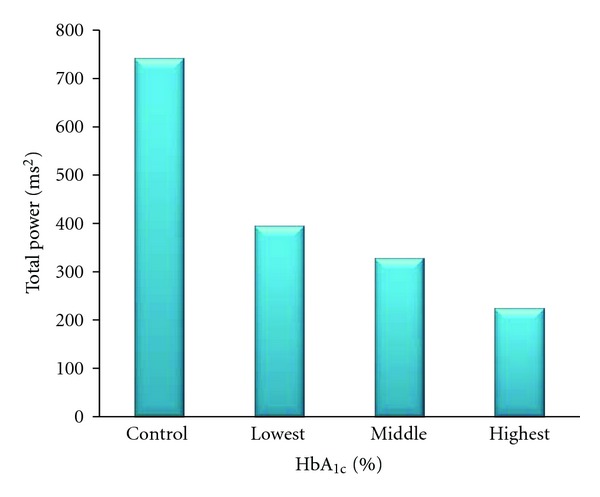
Median of total power in different HbA_1c_ levels.

Figure 1 shows the median values of the total power in normal participants (controls) and diabetics divided by tertiles according to HbA_1c_ levels. We found an inverse association between total power and median HbA_1c_ levels.

## 4. Discussion

We found that substantial autonomic dysfunction was present in our early T2DM patients even before overt clinical symptoms of CVD became apparent. It is noteworthy that involvement of the vagal parasympathetic component of autonomic nervous system was obvious in our T2DM patients. This is evidenced by increased resting heart rate and decreased Valsalva ratio; E/I index and standing ratio in diabetics relative to controls. These findings are in line with those of Freccero et al. who reported a high frequency of parasympathetic and sympathetic neuropathy in both type 1 and type 2 diabetic patients [22]. They suggested that severe damage to large myelinated nerve fibers in addition to the widespread neurological degeneration which usually affects the small nerve fibers of the autonomic nervous system was responsible for profound parasympathetic neuropathy in patients with T2DM. Other researchers also found appreciable degree of autonomic neuropathy in patients with T2DM [23, 24]. 

In fact significantly reduced HRV measures in T2DM patients compared to controls have been previously verified in large-population-based studies [25–27]. In the Hoorn Town of The Netherlands HF, and LF powers and SDNN were lower among T2DM participants compared to those with normal fasting glucose [25]. The same results had also been documented in the Framingham Heart Study and in the Atherosclerosis Risk in Communities (ARIC) cohort [26, 28]. 

The significance of our findings is that HRV reduction in diabetics was present since the early stages of diabetes even before clinical atherosclerotic cardiovascular disease became evident. Thus, it is imperative to screen for autonomic neuropathy as early as possible in T2DM to prevent or retard its serious consequences. 

In addition, we found that surrogate atherosclerosis markers were associated with lower HRV. Especially, increased CIMT in our T2DM participants was significantly associated with decreased HRV. This association was independent of conventional risk factors such as hypertension, BMI, and dyslipidemia. Also, this association was of greater magnitude in diabetic than nondiabetic participants. A stepwise increase in the odds of CIMT with decreasing quartile of total power was observed in those with T2DM.

Gottsäter et al. in a longitudinal study of T2DM patients found a similar correlation between decreasing HRV in the form of total power and increasing CIMT [28]. In their study, this relation was observed both at baseline and with progression of diabetes during 3 years of followup. The association between decreased total power and E/I ratio with increased CIMT has also been observed by Eller et al. previously [29]. In addition, our findings are concordant with those of researchers in the ARIC cohort who had previously reported significant association between lower HRV and coronary heart disease in diabetic subjects [30].

Furthermore, increased CAC score was also associated with lower HRV in our T2DM participants; however, after adjustment for diabetes and other conventional risk factors this relationship lost its significance. Notwithstanding, Rodrigues et al. in their recent study of type 1 diabetes patients found that reduced HRV prospectively predicted progression of coronary artery calcium as a powerful cardiovascular disease risk marker [31]. Previously Colhoun et al. had reported clustering of HRV with coronary calcification and other cardiovascular risk factors in asymptomatic young type 1 diabetes patients [32]. 

We did not find an independent association between reduced HRV and endothelial dysfunction in this study after adjusting for conventional risk factors. However, Pinter et al. in a recent study of young healthy male volunteers found a significant positive correlation between vagal HRV indices (RMSSD, PNN50, and HF power) and normalized flow-mediated dilation [33]. They suggested that endothelial mediators especially nitric oxide released locally from the capillary endothelium could enhance the effects of vagal inputs during respiratory cycle. Vagal discharge increases during expiration and decreases during inspiration, producing respiratory sinus arrhythmia. As a result the major component of short-term HRV which is respiratory related is influenced by endothelial dysfunction.

The association of decreased HRV with atherosclerosis markers could be due to ischemic damage to cardiac nerves [34], which may have been occurred before these markers became apparent. In line with this hypothesis, Gautier et al. in their study of participants in the Pittsburg Study and Kuopio Ischemic Heart Disease Risk Factor Trial found a significant negative relation between mean CIMT and LF power. In other words, LF power, which reflects sympathovagal balance and baroreceptor buffering, was correlated with CIMT. They suggested that decreased LF power could be attributed to chronic downregulation of vascular sympathetic receptors over time. This may potentially cause vascular smooth muscle cell differentiation with subsequent intimal migration and extracellular matrix production [35]. Gottsäter et al. also concluded based on their findings that decreased LF power could predict the progression of atherosclerosis in T2DM [28]. These changes are characteristic of atherosclerotic progression. In our study although LF power was lower in diabetics than controls, this difference hardly became significant (*P* < 0.10). The current study cannot establish this array of events in the course of atherosclerosis development. Further experimental prospective investigations are needed to resolve this cause-and-effect relationship.

Advanced glycation end products may play a critical role in damage to cardiac nerves and subsequent autonomic dysfunction, which promotes progression of atherosclerosis. The inverse association between total power and median HbA_1c_ levels in our study is concordant with this hypothesis. Colhoun and colleagues found that in young type 1 diabetic patients HbA_1c_ was a very good proxy marker of diabetes which could explain differences in HRV indices between diabetics and nondiabetics [32]. On the other hand, Mäkimattila et al. found that total power was the factor most influenced by chronic hyperglycemia in type 1 diabetes patients. In addition, they showed that total power and retinopathy score were the most sensitive measures of diabetes complications [36]. In conclusion, we found that autonomic dysfunction, especially parasympathetic neuropathy, was present since early stages in T2DM. This was strongly related to subclinical atherosclerosis markers in these patients. Specifically decreased total power as a global measure of parasympathic neuropathy was independently associated with increased CIMT.

**Limitations**

A limitation of this study is its cross-sectional nature. So it is not possible to establish a definite cause-and-effect relationship between findings as one is not able to differentiate whether decreased HRV precedes atherosclerosis markers or appears subsequently. In addition, the sample size was relatively limited, and we reported *P* values < 0.10.

## Figures and Tables

**Table 1:** General characteristics of participants.

*n*	Diabetic (*N* = 57)	Control (*N* = 54)
	Mean (SD)	Mean (SD)
Age (year)	50.93 (5.07)	48.73 (5.71)
Female (%)	52.8	58.8
Diabetes duration (years)	8.6 (6.67)	—
Height (cm)	164.36 (8.04)	163.01 (10.24)
Weight (Kg)	73.84 (11.23)	76.26 (11.79)
BMI (Kg/m^2^)	27.37 (3.88)	28.85 (4.79)
WC (cm)	94.18 (7.67)	93.71 (10.46)
FPG (mg/dL)	159.66 (56.76)	94.02 (13.32)^§^
HbA_1c_ (%)	7.95 (1.70)	5.27 (0.67)^†^
Fasting plasma insulin (*μ*IU/mL)	10.29 (6.25)	7.53 (5.03)^†^
HOMA-IR	3.01 (2.38)	2.52 (1.64)^*£*^
Urinary albumin (mg/L)	4.85 (3.16)	3.74 (2.80)
Creatinine (mg/dL)	0.96 (0.17)	0.97 (0.13)
hs-CRP (mg/L)	4.8 (2.3)	3.9 (2.0)^†*£*^
SBP (mmHg)	131.00 (17.55)	120.60 (14.50)*
DBP (mmHg)	77.32 (8.77)	75.71 (10.47)
T-C (mg/dL)	202.27 (31.37)	171.05 (38.45)^†^
HDL-C (mg/dL)	40.69 (8.47)	46.45 (10.86)^†^
LDL-C (mg/dL)	115.08 (22.33)	93.33 (23.48)^†^
TG (mg/dL)	181.91	168.98 (91.14)^*£*^
Metformin (*n*)(%)	43 (76.0)	—
Glibenclamide (*n*) (%)	41 (71.9)	
Statin (*n*) (%)	36 (63.1)	
CIMT (mm)	0.73 (0.17)	0.61 (0.09)^†^
FMD (%)	12.78 (7.54)	18.09 (10.64)^†^
CACS^§^	95.46 (294.12)	24.25 (102.92)^†*£*^

Data are *n*, means ± SD.

WC: waist circumference, FPG: fasting plasma glucose, HOMA-IR: homeostatic model assessment, SBP: systolic blood pressure, DBP: diastolic blood pressure, T-C: total cholesterol, TG: triglyceride, CIMT: carotid intima-media thickness, FMD: (brachial artery) flow-mediated dilation, and CACS: coronary artery calcium.

*P < 0.05, ^†^ *P* < 0.01, and ^§^Agatston score.

^*£*^By Mann-Whitney *U* test.

**Table 2:** Heart rate variability and autonomic function indices.

*n*	Diabetic (*N* = 57)	Control (*N* = 54)
Heart rate (bpm)	73.36 (8.30)	68.94 (9.77)^†^
SDNN (ms)	30.10 (15.43)	40.28 (17.06)^†^
RMSSD (ms)	21.83 (21.20)	31.70 (21.78)*
LFnorm (ms^2^)	55.74 (19.36)	61.98 (20.26)^‡^
HFnorm (ms^2^)	34.01 (20.26)	48.25 (19.36)*
LF : HF ratio	2.19 (2.43)	1.93 (1.77)^‡^
Total power (ms^2^)	1718.34 (337.58)	1156.31 (279.38)^†^
Timed deep breathing	14.6 (8.05)	19.3 (7.26)*
Valsalva ratio	1.24 (0.30)	1.58 (0.28)*
Standing ratio	1.24 (0.20)	1.46 (0.13)^‡^

Data are *n*, means ± SD.

SDNN: standard deviation of the NN intervals; RMSSD: the square root of the mean squared difference of successive NNs.

^‡^ *P* < 0.1, *P < 0.05, and ^†^ *P* < 0.01.

**Table 3:** Partial correlation of total power and coronary risk factors after adjustment for diabetes mellitus.

	Nondiabetic subjects	Diabetic subjects	All subjects
Age (year)	−0.254	−0.099	−0.152
BMI (kg/m^2^)	0.125	−0.230*	−0.130
SBP (mmHg)	−0.020	−0.206	−0.009
DBP (mmHg)	−0.351^†^	−0.291*	−0.319^†^
WC (cm)	−0.095	−0.065	0.070
HDL-C (mg/dL)	−0.131	−0.102	−0.114
LDL-C (mg/dL)	−0.031	0.001	−0.015
Triglyceride (mg/dL)	−0.089	−0.104	−0.100

BMI: body mass index, SBP: systolic blood pressure, DBP: diastolic blood pressure, WC: waist circumference and TG: triglyceride.

*P < 0.05; ^†^ *P* < 0.01.

**Table 4:** Association between categorized CIMT and quartiles of total power in multivariate logistic regression model.

	Unadjusted OR (CI)	Diabetes adjusted OR (CI)	Full adjusted OR (CI)^*ζ*^
Categorized CIMT (<0.7 versus ≥0.7 mm)			

Fist quartile of total power			
Second quartile of total power	0.45 (0.16–0.98)*	0.60 (0.20–1.82)	0.83 (0.23–2.97)
Third quartile of total power	0.24 (0.08–0.71)*	0.31 (0.10–0.96)*	0.23 (0.06–0.89)*
Fourth quartile of total power	0.17 (0.05–0.53)^†^	0.26 (0.08–0.87)*	0.45 (0.09–0.98)*
*P* trends	<0.01	0.01	0.07

Categorized CACS (0–99 versus ≥100^§^)			

Fist quartile of total power			
Second quartile of total power	0.52 (0.18–1.50)	0.73 (0.24–2.27)	1.00 (0.28–3.61)
Third quartile of total power	0.44 (0.15–0.98)*	0.60 (0.19–1.86)	0.76 (0.21–2.71)
Fourth quartile of total power	0.34 (0.11–0.92)*	0.60 (0.18–2.02)	1.42 (0.33–6.04)
*P* trends	0.04	0.37	0.81

Categorized FMD (>8% versus ≤8%)			

Fist quartile of total power			
Second quartile of total power	0.73 (0.21–2.58)	0.86 (0.23–3.11)	0.66 (0.14–3.13)
Third quartile of total power	0.31 (0.07–1.44)	0.39 (0.08–1.88)	0.25 (0.04–1.65)
Fourth quartile of total power	0.34 (0.08–1.40)	0.45 (0.61–5.38)	0.48 (0.08–2.84)
*P* trends	0.31	0.51	0.53

^*ζ*^Full adjustment for age, sex, diabetes mellitus, HDL-C, LDL-C, waist circumference, BMI, and triglyceride in forward conditional model.

^§^Agatston score.

^‡^ *P* < 0.1, *P < 0.05, and ^†^ *P* < 0.01.
